# Are glutathionylated aldehyde reductases the missing piece of the “catecholaldehyde hypothesis” in Parkinson's disease? A medical hypothesis concerning the detoxification of 4-hydroxynonenal (HNE) and 3,4-dihydroxyphenylacetaldehyde (DOPAL)

**DOI:** 10.1016/j.redox.2026.104060

**Published:** 2026-01-27

**Authors:** Rossella Rotondo, Marta Russo, Federico Iacovelli, Valeria Calabrese, Antonio de Iure, Maria Gaglione, Lorenza Leonardi, Gabriella Cocorocchia, Fabrizio Stocchi, Vilberto Stocchi, Maria Francesca de Pandis, Barbara Picconi

**Affiliations:** a Department for the Promotion of Human Sciences and Quality of Life, San Raffaele University, Rome, 00166, Italy; b IRCCS San Raffaele Roma, Via G. Di Biasio 1, Cassino, 03043, Italy; c IRCCS San Raffaele Roma, Rome, 00166, Italy; d Department of Biology, University of Rome Tor Vergata, Via Della Ricerca Scientifica, Rome, 00133, Italy

**Keywords:** DOPAL, HNE, DOPAL-Quinone, GS-DOPAL, Carbonyl reductase 1, Monoamine oxidase, Parkinson's disease, Early biomarker, Mercapturic acids

## Abstract

The autotoxicity of the monoamine oxidase (MAO) reaction product 3,4-dihydroxyphenylacetaldehyde (DOPAL) is central to the “*catecholaldehyde hypothesis”*, which posits that interactions between DOPAL and the protein α-synuclein contribute to the degeneration of catecholaminergic neurons in Parkinson's disease (PD). Dopamine (DA) can undergo spontaneous or enzymatic oxidation, generating dopamine-quinone (DA-Q) and DOPAL, respectively. While growing evidence highlights the quinonization of numerous proteins in catecholaminergic cells due to the high reactivity of DA-Q, the electrophilic properties of DOPAL and its quinone derivative (DOPAL-quinone, DOPAL-Q) have received less attention, along with potential detoxification pathways.

Here, we propose a refinement of the “*catecholaldehyde hypothesis”* by extending the detoxification machinery described for 3-glutathionyl-4-hydroxynonenal (GS-HNE) to the formation of glutathionylated DOPAL adducts. Conjugation of DOPAL-Q with glutathione (GSH) would generate 5-S-glutathionyl-3,4-dihydroxyphenylacetaldehyde (GS-DOPAL). Analogous to GS-HNE, the aldehyde group of GS-DOPAL could be reduced to 5-S-glutathionyl-3,4-dihydroxyphenylethanol (GS-DOPET) by glutathione-dependent aldehyde reductases such as aldose reductase (AKR1B1) and carbonyl reductase 1 (CBR1). Conversely, oxidation of the phenolic hydroxyl groups by CBR1 to yield 5-S-glutathionyl-3,4-dioxophenylacetaldehyde (GS-DOPAL-Q) may also occur. We suggest that the excretion of such GS-adducts via glutathione-electrophile transporters could open new perspectives for identifying early biomarkers of PD and for evaluating the disease-modifying potential of MAO inhibitors.

## Graphical abstract

**Figure:**
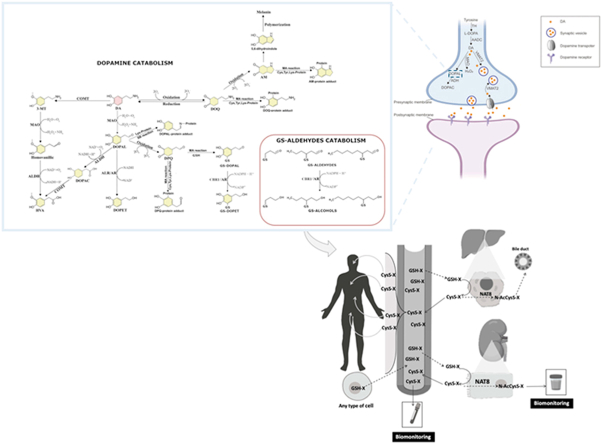

## 1 Introduction

Parkinson's disease (PD) is a chronic, progressive neurodegenerative disorder characterized by both motor and non-motor symptoms [1,2]. While substantial progress has been made in uncovering genetic contributions to PD, the majority of cases (∼90–95 %) are considered sporadic, with only a minority linked to well-established genetic mutations [3].

Among the various pathogenic mechanisms proposed, the dopamine (DA) toxicity hypothesis posits that impaired DA metabolism leads to the excessive formation of reactive oxygen species and electrophilic DA-derived metabolites-such as 3,4-dihydroxyphenylacetaldehyde (DOPAL-) which contribute to neurodegeneration [4]. Elevated intracellular DA levels, its oxidative metabolism, and the formation of reactive quinones are believed to trigger oxidative stress, mitochondrial impairment, and protein dysfunction, as demonstrated in both experimental models and postmortem PD brain tissue [[5], [6], [7], [8]].

Since the introduction of the “*catecholaldehyde hypothesis”* by Goldstein and colleagues [9], attention has increasingly focused on DOPAL, a highly reactive aldehyde produced by monoamine oxidase A (MAO-A)-mediated DA deamination [10]. DOPAL has been implicated in several PD-relevant processes, including α-synuclein aggregation, mitochondrial dysfunction, and selective dopaminergic neuron vulnerability [11,12].

**Fig. 1:**
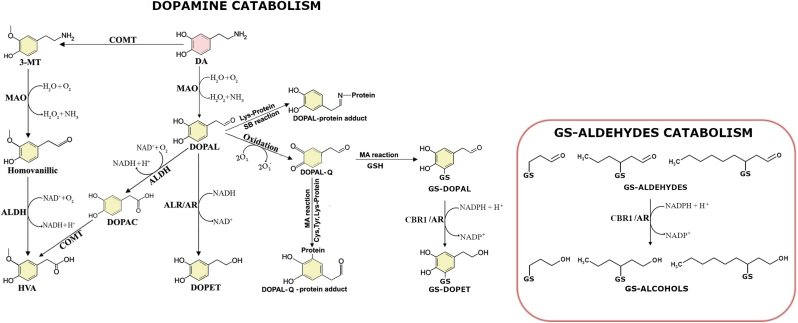
Dopamine catabolism and proposed glutathione-dependent detoxification of DOPAL. The dopamine metabolic pathway was adapted from Zhou et al., 2023 [13]. In the present modification, the DOPAL-quinone (DOPAL-Q) intermediate has been additionally represented as capable of undergoing conjugation with glutathione (GSH), reflecting potential detoxification via glutathionylation. Furthermore, the resulting GS-DOPAL adduct can be reduced to the corresponding alcohol GS-DOPET by enzymes such as carbonyl reductase 1 (CBR1) and aldose reductase (AKR1B1/AR). All other features of the pathway remain as originally depicted.

DA levels are tightly controlled via a homeostatic balance of synthesis, vesicular storage, reuptake, and enzymatic degradation [11]. However, DA can also undergo non-enzymatic (auto-)oxidation or enzymatic oxidation, leading to the generation of dopamine-quinone (DA-Q) and DOPAL, respectively (Fig. 1) [13]. DOPAL is significantly more toxic than DA, with an estimated toxicity 100 to 1000 times greater in both *in vitro* and *in vivo* models [14]. Physiologically, DOPAL concentrations in the substantia nigra (SN) are estimated around 2–3 μM, while levels exceeding 6 μM are associated with pronounced cytotoxicity [15].

Although quinone-mediated protein modification (quinonization) by DA-Q has been extensively studied [16], less is known about the electrophilic properties and protein reactivity of DOPAL-derived quinones (DOPAL-Q), and especially about the cellular mechanisms responsible for their detoxification.

*In vitro*, DOPAL undergoes slow auto-oxidation to form DOPAL-Q. This reaction is significantly accelerated by superoxide dismutase (SOD), used at physiological concentrations (6–7 μM in the cytosol and ∼42 μM in mitochondria) [17]. The presence of SOD reduces the lag phase and increases the rate of DOPAL oxidation, suggesting that DOPAL auto-oxidation generates superoxide anions radicals. Through dismutation, SOD facilitates the accumulation of DOPAL-Q by removing superoxide, thereby shifting the redox equilibrium [18]. Although SOD is generally cytoprotective, its upregulation in the PD SN [19,20] may paradoxically promote the buildup of toxic quinonoid species under pathological conditions.

In addition to SOD, cyclooxygenase-2 (COX-2), an inducible enzyme involved in prostaglandin synthesis, has been implicated in the oxidative metabolism of DA. COX-2 can catalyse the formation of DA-derived semiquinones and quinones that covalently modify nucleophiles including proteins and DNA [21]. Recent evidence has revealed that DOPAL is also a substrate for COX-2, providing an enzymatic pathway for the formation of DOPAL-Q and DOPAL-semiquinones [18]. While Schiff base formation is thought to be crucial for DOPAL's interaction with proteins and the consequent cellular dysfunction, catechol oxidation likely also contributes significantly to DOPAL-induced toxicity [18].

DOPAL detoxification is believed to occur through pathways analogous to those used for other reactive aldehydes, such as 4-hydroxy-2-nonenal (4-HNE). DOPAL is primarily converted to DOPAC by aldehyde dehydrogenases (ALDHs) [22] or to 3,4-dihydroxyphenylethanol (DOPET) via reduction by aldose/aldehyde reductases (e.g., AKR1B1) - as extensively reviewed by Marchitti et al., 2007 [15]. The estimated DOPAL concentration in substantia nigra pars compacta (SNpc) dopaminergic neurons (2–3 μM) is within the range of the K_M_ values reported for these detoxifying enzymes (0.4–1 μM) [15]. However, when DOPAL levels exceed ∼6 μM, toxicity becomes evident in multiple neuronal and non-neuronal cell models [15].

Pioneering work by Burke et al., 2003 [14] demonstrated *in vivo* that direct administration of DOPAL into the rat SN induced more extensive neuronal loss than DA or its major metabolites (DOPAC, DOPET, HVA). Additionally, postmortem studies in patients with sporadic PD have shown accumulation of DOPAL relative to DA in the putamen compared to healthy controls [8], reinforcing the pathological relevance of impaired DOPAL metabolism.

Beyond its aldehyde reactivity, DOPAL's catechol moiety—upon oxidation—facilitates Michael addition reactions, enabling nucleophilic attack by thiol-containing molecules such as cysteine residues and glutathione (GSH) [4]. Thus, DOPAL may not only form Schiff base adducts with lysines, but also undergo glutathionylation, resulting in glutathionylated DOPAL derivatives.

Building on this, we hypothesize a novel detoxification mechanism in dopaminergic neurons involving GSH conjugation of DOPAL-quinone, analogous to the detoxification of 3-glutathionyl-4-hydroxynonanal (GS-HNE) [[23], [24], [25]]. Specifically, DOPAL-Q may react with GSH to form 5-*S*-glutathionyl-3,4-dihydroxyphenylacetaldehyde (GS-DOPAL). The aldehyde group of this conjugate could then be reduced enzymatically to the corresponding alcohol, 5-*S*-glutathionyl-3,4-dihydroxyphenylethanol (GS-DOPET), by enzymes such as aldose reductase (AKR1B1) and carbonyl reductase (CBR1).

This proposed metabolic route provides a plausible mechanism to neutralize DOPAL's dual electrophilicity (i.e., aldehyde and quinone reactivity) through sequential glutathionylation and reduction. It also underscores the potential impact of impaired GSH homeostasis or reductase dysfunction in exacerbating DOPAL toxicity—particularly in the oxidative, inflammatory environment of the PD brain.

## 2 Evaluation of the hypothesis

While the expression of AKR1B1 in dopaminergic neurons—and its involvement in DA metabolism—has been well documented (Fig. 1) [15,26,27], significantly less is known about the presence and functional role of CBR1 in these neurons or in widely used *in vitro* PD models, such as NGF-differentiated PC12 cells. CBR1 is a member of the short-chain dehydrogenase/reductase (SDR) superfamily, broadly expressed across human tissues, and is characterized by a remarkably wide substrate specificity [28]. It catalyses both the reduction of reactive carbonyl species—such as quinones (e.g., 9,10-phenanthrenequinone, menadione), prostaglandins (e.g., PGE_2_), and xenobiotics (e.g., anthracyclines like doxorubicin and daunorubicin) [[29], [30], [31]] — and the oxidation of 15-hydroxyprostaglandins (i.e. PGB1 [32]), among others.

Since its isolation in 1973 from human brain [33], CBR1 has been reported to be implicated in several biological processes, including xenobiotic detoxification, redox regulation, inflammation, and cellular defence against oxidative stress [[34], [35], [36], [37], [38]].

Given its neuroprotective functions [35,39,40] and its substrate overlap with toxic DA catabolites, CBR1 emerges as a plausible candidate for the detoxification of DOPAL-derived quinones, including the putative glutathionylated adduct GS-DOPAL.

### 2.1 Expression of CBR1 in dopaminergic neurons *in vitro*

A critical first step in evaluating this hypothesis is to determine whether CBR1 is expressed in dopaminergic-like cells. This can be assessed using well-established *in vitro* models, including PC12 cells, LUHMES cells, and human iPSC-derived dopaminergic neurons. To the best of our knowledge, direct evidence for CBR1 expression in these cells remains limited. Beyond confirming expression, these models allow investigation of whether CBR1 plays a functional role in neuronal differentiation processes. Given its established roles in redox processes [[41], [42], [43]] and cellular differentiation [44], investigating the presence and regulation of CBR1 in dopaminergic-like cellular models would provide mechanistic insight into how CBR1 might modulate dopamine metabolism, DOPAL detoxification, and neuronal vulnerability, thereby strengthening the relevance of the hypothesis across multiple dopaminergic systems.

To this end, quantitative PCR (qPCR), Western blotting, and immunocytochemistry provide essential tools to assess both the expression levels and subcellular localization of CBR1 in undifferentiated and differentiated dopaminergic-like cells. Although the specific involvement of CBR1 in neuronal differentiation is not fully characterized, several potential roles can be hypothesized based on its biochemical profile:

1. *Protection from oxidative stress:* neuronal differentiation is accompanied by increased oxidative metabolism and vulnerability to reactive oxygen species (ROS) and lipid peroxidation products [45,46]. CBR1 catalyses the detoxification of reactive carbonyl species and their glutathionylated derivatives—including 4-oxo-2-nonenal (4-ONE) and GS-HNE—thereby potentially supporting neuronal survival and maturation [23,47].
2. *Modulation of signaling pathways:* CBR1 is involved in prostaglandin metabolism, including the reduction of prostaglandin E_2_ (PGE_2_), a known regulator of neurite outgrowth and neuroinflammatory processes [48]. Through this activity, CBR1 may indirectly influence differentiation, polarity, and neurotrophic signaling.
3. *Redox homeostasis and transcriptional control:* redox-sensitive transcription factors, particularly Nuclear factor erythroid 2–related factor 2 (Nrf2), orchestrate the expression of antioxidant and cytoprotective genes during neuronal development. Notably, CBR1 is a transcriptional target of Nrf2, and its expression is upregulated in response to Nrf2 activation in multiple experimental contexts, including human-derived cell lines, animal models of oxidative stress and ischemia/reperfusion injury, and human clinical samples [[49], [50], [51], [52]]. This suggests that CBR1 may function both as a modulator of the intracellular redox environment and as an effector of the Nrf2-dependent cytoprotective response. Although direct evidence in dopaminergic neurons remains limited, Nrf2 activation could plausibly enhance CBR1-mediated detoxification of electrophilic DOPAL-derived species, thereby mitigating catecholaldehyde-induced stress during neuronal differentiation and under PD-relevant oxidative conditions.

### 2.2 Exploring GS-DOPAL as a substrate for CBR1 and AKR1B1: Computational and experimental approaches

Despite the rationale supporting a role for CBR1 in DOPAL detoxification, no direct evidence currently confirms that GS-DOPAL is a substrate for either CBR1 or AKR1B1. Nonetheless, both enzymes are well-established in catalyzing the reduction of glutathionylated aldehydes derived from lipid peroxidation, suggesting a potential mechanistic link to GS-DOPAL metabolism. Aldose reductase (AR) exhibits markedly higher catalytic efficiency toward glutathione conjugates of acrolein, *trans*-2-hexenal, *trans*-2-nonenal, and trans,*trans*-2,4-decadienal, with activities ranging from 4- to 1000-fold greater than those observed for the corresponding free alkanals. Structural modifications of the glutathione moiety attenuate AR-mediated reduction of the acrolein adduct, consistent with specific interactions between glutathione residues and the enzyme active site [53]. In parallel, human CBR1 displays minimal reactivity toward a series of free alkanals and alkenals; however, the introduction of a glutathionyl group, as in GS-propanal, GS-hexanal, and GS-nonanal, converts these molecules into highly efficient substrates, highlighting the enzyme's specialized role as a glutathionylated-aldehyde reductase. Furthermore, substrate hydrophobicity appears to modulate catalytic performance, with GS-nonanal, structurally most similar to the open aldehyde form of GS- HNE, exhibiting the highest affinity and specificity constant (k_cat_/K_M_), while GS-hexanal and GS-propanal display proportionally lower catalytic efficiencies [23].

Given the chemical structure of GS-DOPAL, a glutathionylated catecholaldehyde, it is plausible that this adduct could interact with the active sites of CBR1 or AKR1B1, although its enzymatic reduction has not been experimentally demonstrated.

**Fig. 2:**
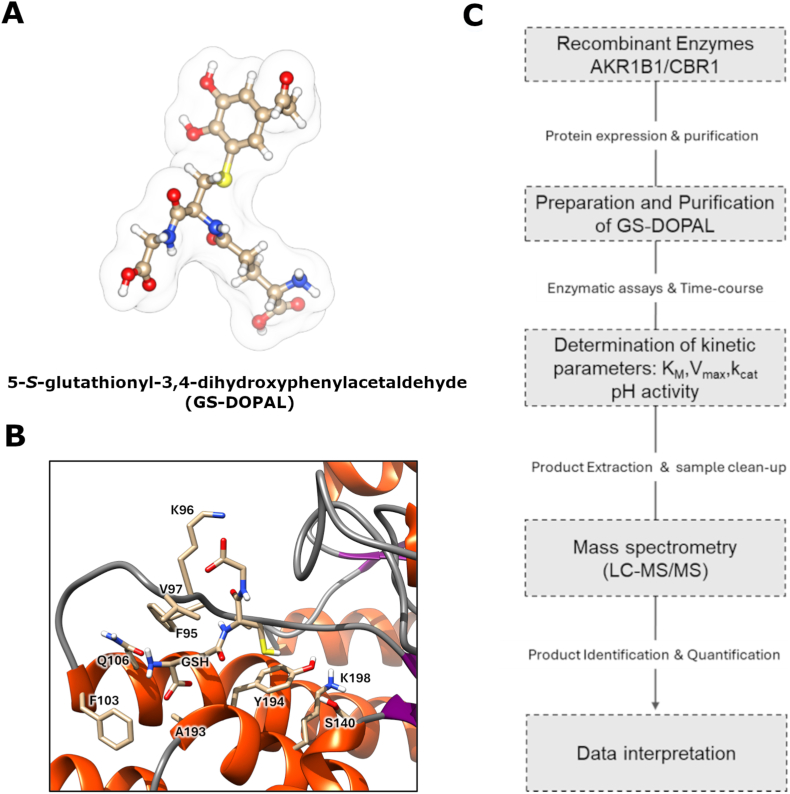
Dual-pronged approach to study GS-DOPAL conversion into GS-DOPET: (A) 3D structure of GS-DOPAL; (B) Crystal structure of human Carbonyl Reductase 1 (PDB 3BHJ) in complex with GSH, highlighting the active site; (C) Experimental workflow: from expression and purification of the recombinant enzyme to *in vitro* enzymatic assay and identification of reaction products by LC-MS/MS.
• In vitro enzymatic assays using purified recombinant human CBR1 and AKR1B1, coupled with LC-MS or HPLC analysis, should be performed to monitor the conversion of GS-DOPAL into its reduced counterpart, GS-DOPET (Fig. 2C).

To fill this knowledge gap, we propose a dual-pronged experimental approach:

• AI-based structural predictions combined with molecular dynamics (MD) simulations could yield structural and dynamical insights into the interaction of GS-DOPAL with the active sites of these enzymes, thus formulating assessable mechanistic hypotheses (Fig. 2A and B)

**Fig. 3:**
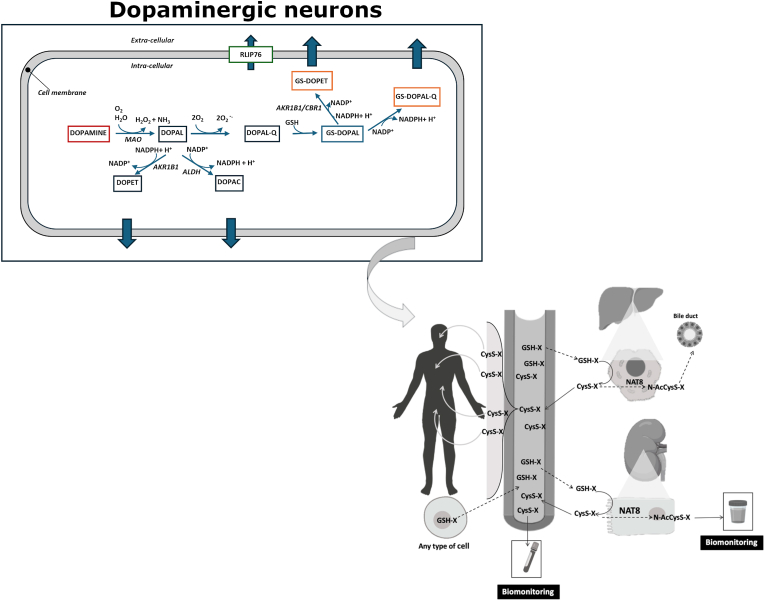
Integrated view of DOPAL biotransformation and the mercapturic acid pathway. DOPAL can be conjugated with glutathione (GSH) and extruded from the cell. GSH-S-conjugates circulate and are metabolized primarily at the apical membrane of kidney proximal tubular cells and, to a lesser extent, hepatocytes. The resulting cysteine-S-conjugates (Cys-S-conjugates) can be further detoxified by N-acetyl-transferase 8 (NAT8) to form mercapturates (N-AcCysS-X) that are excreted in urine, or reabsorbed and distributed to other organs. Blood and urine can serve as matrices for biomonitoring mercapturate pathway metabolites. Abbreviations: MAO: monoamino oxidase; ALDH: aldehyde dehydrogenase; AKR1B1: aldose reductase; CBR1: Carbonyl Reductase 1; CysS-X: cysteine-S-conjugates; GSH-X: glutathione-S-conjugates; N-AcCysS-X: mercapturates; NAT8: N-acetyl-transferase 8. Adapted from Gonçalves-Dias C et al., 2019 [55].

However, in addition to its reductive activity, CBR1—belonging to the short-chain dehydrogenase/reductase (SDR) superfamily—is also capable of catalyzing oxidative reactions. Notably, GS-HNE exists predominantly as a cyclic hemiacetal in aqueous solution (∼95 %) [54], and CBR1 oxidizes the hydroxyl group of this form to produce specifically γ-lactone GS-HNA [23,24]. By analogy, it is conceivable that the catecholic hydroxyls of GS-DOPAL could undergo NADP^+^-dependent oxidation by CBR1, potentially leading to the formation of 5-*S*-glutathionyl-3,4-dioxophenylacetaldehyde (GS-DOPAL-Q), a putative quinonoid metabolite with increased electrophilic reactivity (Fig. 3).

This raises the possibility of a dual metabolic fate for GS-DOPAL: enzymatic reduction to GS-DOPET or oxidative conversion to GS-DOPAL-Q. The latter species, if formed, may exhibit distinct redox or toxicological properties, and its existence would further implicate CBR1 in both detoxification and redox signaling pathways relevant to catecholaldehyde stress. This hypothesis warrants direct biochemical testing to determine the preferred catalytic route under physiologically relevant conditions.

### 2.3 Efflux of glutathione–DOPAL conjugates: a proposed detoxification pathway in dopaminergic neurons

Although direct experimental evidence for GS-DOPAL efflux in dopaminergic neuronal cell models is currently lacking, established mechanisms governing the export of glutathione conjugates provide a strong conceptual framework to explore this pathway in PD-relevant cellular systems. As observed for glutathionylated lipid peroxidation products such as GS-HNE, many GS- conjugates—derived from both endogenous aldehydes and xenobiotics—are actively exported from cells and subsequently metabolized to mercapturic acids via the mercapturic acid pathway [[56], [57], [58]]. In this context, elucidating the formation and extracellular release of the GS-DOPAL in *vitro* dopaminergic neuronal cell models exposed to mitochondrial toxins (e.g., rotenone or MPTP) or directly to DOPAL, could provide critical insights into neuroprotective detoxification mechanisms under PD-relevant oxidative stress conditions.

A systematic analysis combining quantitative profiling of extracellular metabolites with pharmacological inhibition or genetic knockdown of key efflux transporters [59] would enable a detailed characterization of the mechanisms governing GS-DOPAL export and clarify its role in cellular defense pathways.

The conjugation of DOPAL-derived quinones with GSH and their subsequent export is likely mediated by glutathione S-transferases (GSTs) and ATP-binding cassette (ABC) transporters, such as multidrug resistance-associated proteins (MRPs), which facilitate the efflux of GSH conjugates [59]. Alterations in this process—due to GSH depletion, reduced GST activity, or transporter dysfunction—could compromise the cell's capacity to neutralize reactive dopamine metabolites, thus increasing vulnerability to catecholaldehyde-induced stress. This is particularly relevant to PD etiopathogenesis, where oxidative stress, impaired detoxification of reactive aldehydes such as DOPAL, and dysregulated GSH metabolism are key contributors to dopaminergic neurodegeneration [4,60,61]. Supporting this view, GSH and cysteine conjugates of dopamine—such as 5-*S*-glutathionyl-dopamine and 5-*S*-cysteinyl-dopamine—have been detected in the cerebrospinal fluid (CSF) of PD patients, indicating that similar conjugation and efflux mechanisms operate in the human brain under pathological conditions [62]. Importantly, DOPAL itself has been reported to irreversibly inhibit GST activity [63], suggesting that excessive intracellular DOPAL accumulation could impair the conjugation and detoxification of multiple electrophilic species. Therefore, the presence of enzymes capable of metabolizing GS-DOPAL, such as CBR1 or AKR1B1, may not only prevent intracellular accumulation of this glutathionylated adduct but also contribute indirectly to maintaining low steady-state levels of free DOPAL, thereby preserving GST function and overall cellular detoxification capacity.

Detection of GS-DOPAL or its downstream metabolites in the extracellular medium of dopaminergic neuronal models would thus provide a functional readout of detoxification efficiency and intracellular redox balance (Fig. 3). High-performance liquid chromatography coupled with electrochemical detection (HPLC-ECD) remains the gold standard for detecting catechols such as DOPAL and its metabolites due to its high sensitivity and selectivity [4]. In parallel, liquid chromatography-tandem mass spectrometry (LC-MS/MS) offers the specificity required for the detection and quantification of complex GSconjugates. This approach has been successfully applied to analyze GS-HNE and its oxidative/reductive metabolites, and can be similarly adapted for the identification and characterization of GS-DOPAL and its related derivatives [64]. Time-course studies employing these analytical techniques, in conjunction with pharmacological modulation of GST or MRP activity, could clarify the kinetics of GS-DOPAL formation, the enzymatic pathways involved in its redox transformation, and the mechanisms governing its export from cells.

### 2.4 Potential of GS-DOPAL-derived mercapturic acids as biomarkers for early Parkinson's disease

The detection of mercapturic acid (MA) derivatives of GS-DOPAL and its downstream catabolic products in biological fluids, such as urine or plasma, represents a promising avenue for early biomarker discovery in PD (Fig. 3). Elevated levels of these metabolites may reflect increased oxidative stress or impaired detoxification pathways, potentially manifested as higher concentrations of mercapturic acid conjugates derived from DOPAL and DOPET.

Systematic monitoring of these metabolites may provide a non-invasive readout of intracellular catecholaldehyde stress and detoxification efficiency, potentially enabling the detection of PD-related biochemical changes prior to overt dopaminergic neurodegeneration. Integrating these measurements with complementary *in vitro* studies of GS-DOPAL formation, enzymatic reduction by CBR1 and AKR1B1, and efflux via GSTs and MRPs could further strengthen their utility as early indicators of cellular redox imbalance and neuroprotective capacity.

Since DOPAL can inhibit ALDH [60], its detoxification via GSH conjugation—and the consequent increase in mercapturic acid derivatives of DOPAL (MA-DOPAL) and related metabolites—may reflect a functional interplay between DOPAL and HNE metabolism. Both aldehydes compete for detoxification through ALDH and glutathione-dependent pathways, suggesting that alterations in MA-DOPAL levels could serve as indicators of a broader oxidative stress profile involving lipid peroxidation. Consequently, mercapturic acids derived from GS-DOPAL and its metabolites represent promising candidates for non-invasive, early biomarkers of PD, particularly as indicators of dopamine metabolism-related oxidative stress. Nonetheless, further clinical validation—including determination of diagnostic cut-off values, patient stratification, and consideration of oxidative stress-related comorbidities—is essential to substantiate this hypothesis for diagnostic application.

### 2.5 From bench to bedside: could we repurpose MAO inhibitors as disease-modifying therapies?

MAO inhibitors (MAOIs) are commonly used in combination with Levodopa in PD, where they prolong dopamine availability and improve motor control by reducing its enzymatic breakdown. However, emerging evidence suggests that their therapeutic potential may extend beyond symptomatic relief. From a mechanistic standpoint, early MAO inhibition could limit theoretically the formation and accumulation of DOPAL, a highly reactive dopamine metabolite implicated in selective dopaminergic neurodegeneration [65]. This concept is supported by the PDRisk study, which reported that individuals with multiple PD risk factors and reduced CSF levels of DA precursors and metabolites (notably DOPA and DOPAC) exhibited a higher probability of developing clinically manifest PD within a few years. These observations highlight the value of neurochemical biomarkers of central DA deficiency—detectable even before motor onset—as potential indicators for early neuroprotective intervention, mitigating oxidative and aldehyde-induced neurotoxicity [66]. Nevertheless, translating this concept into effective clinical strategies remains challenging. The PDRisk study is relatively small and highly selected cohort limits generalizability to broader populations with fewer or different risk factors. Furthermore, CSF collection is invasive, emphasizing the need for non-invasive surrogate biomarkers, such as urinary or plasma mercapturic acid derivatives of DOPAL, that could enable patient stratification and monitoring of therapeutic efficacy.

Mechanistic studies support a DOPAL-targeted approach. MAO inhibition effectively reduces DOPAL formation [66]. However, clinical experience with MAOIs has yielded disappointing disease-modifying outcomes. As highlighted by Khashab and colleagues [67], a key explanation may lie in the altered handling of DA in PD. Impaired vesicular storage leads to an abnormal accumulation of cytosolic DA, which undergoes spontaneous oxidation to neurotoxic quinones leading to the formation of aminochrome and 5-*S*-cysteinyl-dopamine (cys-DA) [[68], [69], [70]]. Consequently, while MAOIs reduce DOPAL levels, they may simultaneously promote cytosolic DA oxidation, thereby attenuating their overall neuroprotective benefit—a trade-off effect. This phenomenon has been demonstrated in PC12 cells treated with MAOIs, where DOPAL reduction was accompanied by an increase in cys-DA [71]. Importantly, co-administration of the antioxidant N-acetylcysteine (NAC) prevented this effect. Building on these findings, combined treatment with MAOIs and NAC in preclinical models further enhances DOPAL suppression and mitigates oxidative stress [67], likely through NAC's ability to cross the blood–brain barrier and restore intracellular glutathione [72,73].

Another critical factor identified by Khashab and colleagues [67] is the timing of intervention. Most clinical trials to date have enrolled symptomatic patients, often years after diagnosis, when 70–80 % of substantia nigra dopaminergic neurons have already degenerated. At this stage, available therapies remain primarily symptomatic, leaving neurodegeneration unchecked. Patients consequently experience progressive motor deficits, often requiring escalating support from caregivers and specialized rehabilitation services to maintain functional independence and quality of life. These limitations highlight the urgent need for early detection and intervention strategies aimed at preserving neuronal integrity before significant motor impairment occurs.

Mathematical modeling of PD pathogenesis suggests that targeting the preclinical phase could substantially delay symptom onset [74]. Early initiation of MAOI-based therapy—particularly in biomarker-defined at-risk populations—may therefore reveal disease-modifying effects that were missed in trials conducted at advanced stages. In this context, monitoring MA-DOPAL levels could serve as a valuable pharmacodynamic biomarker to assess the efficacy of MAOIs, either as monotherapy or in combination with glutathione-boosting agents.

Future research should prioritize longitudinal and drug-repositioning studies in prodromal or at-risk populations, using biomarker endpoints such as MA-DOPAL and its derivatives in plasma or urine. Careful control for confounding factors—including smoking status, comorbid oxidative stress-related conditions, and relevant genetic variants—will be critical to ensure validity.

Ultimately, the identification of reliable, non-invasive biomarkers combined with the repurposing of well-characterized agents with potential disease-modifying effects offers a promising strategy. Such approaches could preserve dopaminergic neurons, delay the onset of clinical symptoms, and reduce the long-term burden on healthcare systems—shifting the therapeutic paradigm toward early, mechanism-based neuroprotection.

## 3 Conclusions

This hypothesis-driven work integrates dopamine metabolism, redox biology, and GSH-dependent detoxification to propose a previously unrecognized extension of the catecholaldehyde hypothesis of PD, linking the detoxification pathways of lipid peroxidation–derived aldehydes such as HNE with dopamine-derived catecholaldehydes such as DOPAL. Specifically, we propose that the quinonoid form of DOPAL may undergo GSH conjugation, generating GS-DOPAL as a key intermediate in a protective detoxification cascade. By drawing parallels with the well-characterized metabolism of glutathionylated lipid peroxidation products, we propose CBR1 and AKR1B1 as plausible enzymatic mediators of GS-DOPAL reduction, thereby neutralizing both aldehydic and quinonoid reactivity. Although direct experimental evidence for GS-DOPAL formation and metabolism in dopaminergic neurons is currently lacking, the substrate specificity of CBR1 and AKR1B1 toward other GS-aldehydes strongly supports their potential involvement in this pathway, which will be further validated using molecular docking and *in vitro* enzymatic assays.

The proposed export of GS-DOPAL via GST- and MRP-dependent mechanisms and its subsequent processing through the mercapturic acid pathway provides a conceptual framework linking intracellular catecholaldehyde stress to measurable extracellular and systemic metabolites. In this context, mercapturic acid derivatives of DOPAL emerge as promising non-invasive biomarkers reflecting dopamine-related oxidative stress and detoxification capacity—features that are particularly relevant for early or prodromal stages of PD.

Finally, by integrating this biochemical framework with clinical observations and therapeutic considerations, we suggest that DOPAL-centered pathways may help explain both the potential and the limitations of MAO inhibition as a disease-modifying strategy. The effectiveness of such interventions is likely to depend critically on timing, redox context, and the capacity of complementary detoxification systems, including GSH homeostasis.

Overall, this work provides a unifying mechanistic model that connects dopamine metabolism, redox regulation, enzymatic detoxification, biomarker discovery, and therapeutic timing. Experimental validation of GS-DOPAL formation, metabolism, and efflux in dopaminergic models will be essential to substantiate this hypothesis. If confirmed, these pathways could open new avenues for mechanism-based neuroprotection and for the development of early, non-invasive biomarkers to guide intervention before irreversible dopaminergic neurodegeneration occurs.

## CRediT authorship contribution statement

**Rossella Rotondo:** Conceptualization, Writing – original draft, Writing – review & editing. **Marta Russo:** Conceptualization, Writing – original draft. **Federico Iacovelli:** Conceptualization, Writing – original draft. **Valeria Calabrese:** Conceptualization. **Antonio de Iure:** Writing – original draft. **Maria Gaglione:** Conceptualization. **Lorenza Leonardi:** Conceptualization. **Gabriella Cocorocchia:** Conceptualization. **Fabrizio Stocchi:** Writing – review & editing. **Vilberto Stocchi:** Writing – review & editing. **Maria Francesca de Pandis:** Conceptualization, Writing – original draft, Writing – review & editing. **Barbara Picconi:** Conceptualization, Writing – original draft, Writing – review & editing.

## Funding

This work was supported by funding of the Italian Ministry of Health [Ricerca Corrente].

## Declaration of competing interest

None.

## Data Availability

No data was used for the research described in the article.
